# The Epidemiology and Clinical Burdens of Human Parainfluenza Virus Infections Amongst Hospitalized Children Under 5 Years of Age in Jordan: A National Multi-Center Cross-Sectional Study

**DOI:** 10.3390/v17020170

**Published:** 2025-01-25

**Authors:** Munir Abu-Helalah, Mohammad Abu Lubad, Mohammad Al-Hanaktah, Ahmad Al Tibi, Maisalreem Alhousani, Simon B. Drysdale

**Affiliations:** 1 Department of Family and Community Medicine, Faculty of Medicine, University of Jordan, Amman 11942, Jordan; 2 Public Health Institute, University of Jordan, Amman 11942, Jordan; 3 Department of Microbiology and Pathology, Faculty of Medicine, Mutah University, Karak 61710, Jordan; abu_lubbad@mutah.edu.jo; 4 Faculty of Medicine, University of Jordan, Amman 11942, Jordan; malhanaktah49@gmail.com; 5 Molecular Genetics Supervisor, Biolab Diagnostic Laboratories, P.O. Box 5153, Amman 11183, Jordan; a.tibi@biolab.jo; 6 Research Assistant, Mutah University, Karak 61710, Jordan; maishousani@gmail.com; 7 13 Oxford Vaccine Group, Department of Pediatrics, University of Oxford, Oxford OX1 2JD, UK; simon.drysdale@paediatrics.ox.ac.uk; 8 The NIHR Oxford Biomedical Research Centre, Oxford OX3 7JX, UK

**Keywords:** human parainfluenza virus, clinical, epidemiological, children, below age of five, Jordan

## Abstract

Human parainfluenza virus (HPIV) is a major cause of respiratory illnesses in children under five years, with clinical manifestations ranging from mild upper respiratory tract infections to severe lower respiratory tract diseases. This multi-center, cross-sectional study investigated the burden, clinical features, and predictors of respiratory viral infections in hospitalized children across four sites in Jordan. Nasopharyngeal specimens from 1000 eligible children were analyzed. In this article, we focused on HPIV infections. The overall HPIV positivity rate was 22.6%, with HPIV-3 accounting for 90.3% of cases. Significant geographic variability was observed, with higher positivity rates in the southern region. HPIV-positive cases frequently presented with symptoms like nasal congestion, tachypnea, and poor feeding. Co-infections with respiratory syncytial virus (RSV) or influenza were associated with worse outcomes, including an increased need for invasive ventilation. The logistic regression identified male gender, asthma, and respiratory acidosis as predictors of complications. Geographic differences in HPIV prevalence and severity were notable, emphasizing the influence of environmental and socioeconomic factors. These findings underscore the urgent need for enhanced HPIV surveillance, targeted public health interventions, and vaccine development to mitigate the disease burden. This study provides critical insights that guide healthcare strategies and improve outcomes in young children at risk of severe HPIV infections.

## 1. Introduction

Human parainfluenza viruses (HPIVs) are a well-established cause of respiratory illnesses in children and adults with a wide range of clinical manifestations, including mild upper respiratory tract infections, croup, bronchiolitis, and pneumonia [1].

HPIV has four major serotypes (HPIV-1 to HPIV-4) with varying global rates of infection. HPIV usually causes epidemics in the spring and early summer in the northern hemisphere. HPIV can cause lower respiratory tract infections and more severe infections in young children [2,3].

A systematic review on HPIV-associated acute lower respiratory tract infection (ALRI) published in 2021 identified 203 studies, including 162 published studies and a further 41 high-quality unpublished studies. The review found that HPIV caused an estimated 18.8 million cases of ALRI globally in 2018 and caused 725,000 (433,000–1,260,000) ALRI hospital admissions. The study also revealed that there were 34,400 (16,400–73,800) ALRI deaths attributable to HPIVs among children younger than 5 years during the same period [4].

A recent study from Zhejiang, China, that included 7072 samples from children younger than 18 years showed an overall HPIV positivity rate of 6.9%. The common clinical manifestations were fever (93.4%, 456/488), cough (94.7%, 462/488), wheezing (26.2%, 128/488), and shortness of breath (14.8%, 72/488). In total, 213 (43.6%) cases had co-infection, and 138 (28.3%) cases had extrapulmonary symptoms. Interestingly, 19.7% of the participants experienced intrapulmonary and intrathoracic complications [5].

An analysis of viral swab data from Al Bashir Hospital, the largest public hospital in Jordan, for the years 2010 to 2013 revealed a HPIV positivity rate of 7% [6]. There is a need for updated data on the HPIV positivity rates in Jordan, particularly in the post COVID-19 pandemic era, from representative sites. There is also a need for the assessment of clinical manifestations and complications to assess the burden of HPIV in Jordan and to compare the results for different HPIV serotypes (1, 2, 3, and 4). This has been achieved through a further analysis of samples, as described below, collected for a study on the burden of pediatric respiratory infections in Jordan for the period between November 2022 and April 2023. The first published report from this study revealed a high burden of Respiratory Syncytial virus (RSV) in Jordan, with positivity rates reaching 50.6% [7]. This report will examine the burden of HPIV and evaluate the role of RSV and influenza coinfections in clinical presentations and complications.

## 2. Materials and Methods

### 2.1. Study Design

The multi-center cross-sectional study design involved four study centers distributed in the central, northern, and southern regions of Jordan as follows: (1) Princess Rahma Hospital for Children, Irbid; (2) Zarqa Hospital, Al Zarqa, which serves the center and eastern of Jordan; (3) Jordan University Hospital, Amman; and (4) Al Karak Public Hospital, Al Karak.

The full methods were described previously by Abu-Helalah et al. [7]. In brief, children <5 years old who presented with respiratory symptoms to the inpatient clinics at the four study sites were recruited. The recruitment of patients took place during weekdays and continued during the weekends and holidays to enhance the diversity of enrollment.

### 2.2. Sample Collection and Processing

- Nasopharyngeal (NP) specimens were collected from each patient who met the inclusion criteria and consented to the study. An NP swab was taken and then a Multiplex viral reverse transcription polymerase chain reaction (RT-PCR) was performed on each nasopharyngeal specimen. Samples were initially analyzed for RSV and influenza infections, and then stored at −80 °C, as described below, for further analysis [7].
- Polymerase chain reaction (PCR) was subsequently used to diagnose cases with Human Parainfluenza Virus (HPIV) at the included sites.
- HPIV-positive samples were further analyzed via subtyping for HPIV-1, HPIV-2, HPIV-3 and HPIV-4.

### 2.3. Microbiology—Sample Collection and Transport

This study is based on a further analysis of previously collected samples, which were stored at −80 °C using TSX Universal Series ULT freezers (Thermo Fisher Scientific^®^, Waltham, MA, USA) until further analysis.

As described previously [7], nasopharyngeal specimens were collected from eligible patients. Samples were processed immediately to ensure RNA integrity.

RNA extraction was performed using the Zybio-Nucleic Acid Isolation System EXM 3000 (Zybio Inc., Chongqing, China) according to the manufacturer’s protocol. Extracted RNA was aliquoted and stored at −80 °C using TSX Universal Series ULT freezers (Thermo Fisher Scientific^®^, Waltham, MA, USA) until further analysis. Storage was for 18 months. For the detection of human parainfluenza virus (HPIV) and its subtypes HPIV-1, HPIV-2, HPIV-3, and HPIV-4, the VIASURE Respiratory Panel III (Certest Biotec, S.L., Zaragoza, Spain) was used, as well as a multiplex real-time reverse transcription polymerase chain reaction (RT-PCR) assay. Amplification and analysis were performed using the QuantStudio™ 5 Real-Time PCR System, 96-well, 0.2 mL (Applied Biosystems^®^, Foster, CA, USA). The PCR cycling conditions were as follows: reverse transcription at 45 °C for 15 min, initial denaturation at 95 °C for 2 min, followed by 45 cycles of denaturation at 95 °C for 10 s and annealing/extension at 60 °C for 50 s.

### 2.4. Power/Sample Size

We planned to enroll 1000 subjects who matched the above clinical criteria. According to previous studies, the overall HPIV detection rate of 6.15%, varying from 5.04% in 2022 to 9.70% in 2020. Another study from Sri Lanka showed a positivity rate of 9.4% [8,9]. Moreover, based on previous data from Jordan, the positivity rate for HPIV was 7%. Therefore, it was expected that 7% to 9% of the subjects would be positive for HPIV when calculating the sample size [6].

### 2.5. Statistical Methods

Statistical Package for the Social Sciences (SPSS) version 23 was used to analyze the data. Descriptive statistics (Student’s *t*-test and chi-squared test) were used to analyze and compare the categorical variables: demographic characteristics, including the patient characteristics; risk factors; pre-hospital antibiotic use; and vaccination details. Logistic regression analysis was used to identify the predictors of HPIV positivity and predictors of complications.

### 2.6. Case Report Form (CRF)

This was described previously [7]. In brief, each study participant was assigned a unique case ID for the duration of the study. The first part of the form included the inclusion criteria for this study, as previously described. The parents/guardians of the eligible patients were asked if they consented to their child taking part in the study after explaining the details.

### 2.7. The Interview Forms Included Five Sections

1. Background, demographic, and societal data for patients and parents: sex, age, parents’ age and educational status, special diet/milk, number of people in the household with age groups, patients or siblings attending a kindergarten, and parents or siblings with a history of asthma or eczema. Furthermore, a detailed smoking history was obtained, including the mother’s history of smoking during pregnancy; the father’s, mother’s, or other household members’ smoking (inside or outside the home); and the number of cigarettes per day or waterpipe per week.
2. Medical history, including birth history, existing medical conditions, and current regular medications.
3. Presenting symptoms and signs: Symptoms and their duration prior to admission were included, as well as other clinical manifestations, such as cardiovascular manifestations, dehydration, wheezes, cyanosis, low activity level, hypoxia (SaO_2_ < 92%), tachypnea, pneumothorax/atelectasis, apnea > 10 s, subcostal/intercostal retractions, and nasal flaring.
4. Laboratory findings were included, such as the patients’ white blood cell (WBC) count and differential, blood gas, PCR results, chest X-ray findings on arrival, pharyngeal swab, bacterial coinfection, fungal coinfection, and others.
5. Healthcare prior to hospitalization at public or private hospitals and clinics and financial cost of the provided care: This included care provided at the emergency department at the same hospital prior to admission.

All medicines utilized during admission were recorded for every patient. This included all discharge medications.

## 3. Results

### 3.1. Inpatient Data

During the recruitment phase between 15 November 2022 and 14 April 2023, there were 3580 hospitalizations at the study sites. According to the study eligibility criteria, 1755 children were screened; of them, 1008 were eligible for the study. Only eight participants were not included due to the failure of their parents to sign the consent form. A total of 1000 individuals who met the criteria were therefore enrolled in this study.

The mean age of all the participants was 17.10 (SD: 16.57) months and the median age was 9.68 (Q1–Q3: [3.13–29.83]) months. Meanwhile, the mean age for HPIV-positive cases was 21.65 ± 1.15 months compared with 15.77 ± 0.58 for HPIV-negative cases (*p*-value < 0.001). Approximately 60.18% of HPIV-positive cases were male; however, 58.14% of HPIV-negative cases were male. In addition, 29.65% of the HPIV-positive cases were younger than 6 months; however, this value was 40% for those negative for HPIV.

**Figure 1:**
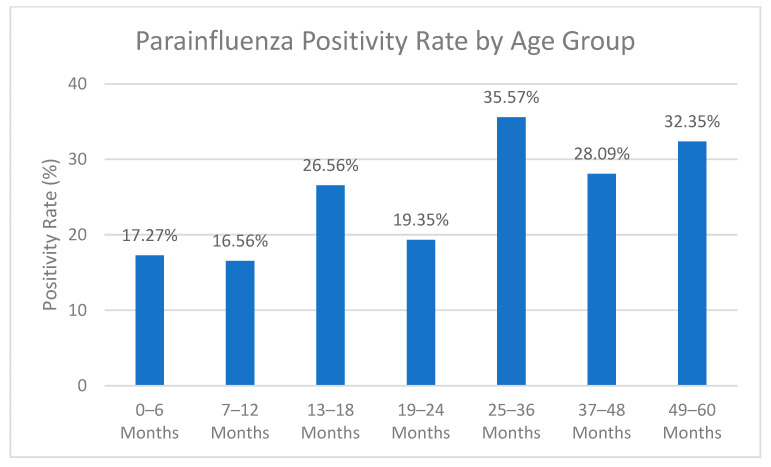
Parainfluenza positivity rate by age group (15 November 2022–14 April 2023). *x*-axis: Positivity Rate (%). *y*-axis: Age in Months.

The key finding of this study was the HPIV positivity rate of 22.6% (*n* = 226), with a predominance of HPIV-3. The positivity rates for HPIV 1, 2, 3, and 4 were 5.3%, 2.7%, 90.3%, and 3.5%, respectively. The rate of coinfections between HPIV types was only 1.7%. The highest positivity rates were reported for children 25 to 36 months old, followed by those 49 to 60 months old. The lowest positivity rates were observed in children 7 to 12 months old (Figure 1).

The positivity rates by month of admission in the recruitment period are shown in Supplementary Table S1. The highest positivity rate was reported in January (*n*_positive_ = 54, 36.49%) followed by December (*n*_positive_ = 144, 31.58%), while the lowest positivity rates were reported in November (*n*_positive_ = 13, 4.06%).

When comparing the demographic factors and medical histories of the HPIV-positive and HPIV-negative individuals (Supplementary Table S2), several significant disparities became known. The positivity rate was lower in participants 6 months old or younger than in older participants (*p*-value = 0.005). The highest positivity rate was in Karak city (29.60%), which is in the southern region of Jordan (*p*-value < 0.001).

The data provided in Table 1 illustrate the distribution of various symptoms across the study subjects. It was observed that the HPIV-positive cases had statistically higher rates of sore throat (*p*-value = 0.041), rhinorrhea (*p*-value < 0.001), nasal congestion (*p*-value < 0.001), poor feeding (*p*-value = 0.002) and tachypnea (*p*-value < 0.001) compared with those who tested negative for HPIV. Interestingly, cases coinfected with RSV or influenza had statistically higher rates of rhinorrhea, nasal congestion and tachypnea than those infected with HPIV only (*p*-value = 0.001, *p*-value < 0.001, and *p*-value = 0.001, respectively), as shown in Table 2. On the other hand, those positive for HPIV only had higher rates of both sore throat and poor feeding (*p*-value = 0.015, and *p*-value = 0.006, respectively) (Table 2). The duration of the reported rhinorrhea, nasal congestion and tachypnea was statistically significantly higher for HPIV-positive cases when compared with HPIV-negatives cases (Table 3).

The investigations during hospitalization and clinical signs and outcomes are shown in Table 4 and Table 5. Based on the clinical assessment, clinical signs such as wheezing and tachypnea were significantly higher among the HPIV-positive patients than the HPIV-negative patients (*p*-value = 0.032, and *p*-value = 0.036, respectively). Interestingly, the need for invasive ventilation was higher in those co-infected with RSV or influenza than those infected with only HPIV or those negative for HPIV (*p*-value = 0.045)**.** However, dehydration as a complication was more prevalent among the HPIV-negative patients than the HPIV-positive patients (*p*-value = 0.009), and was higher among those with a coinfection compared with the ones with only HPIV (*p*-value = 0.019) (Table 4). However, there were no statistically significant differences in the complication rates between HPIV-positive cases according to the presence of influenza or RSV coinfection, except for the need for invasive ventilation (*p*-value = 0.05) (Table 5).

### 3.2. Predictors of HPIV Positivity

The results of the logistic regression analysis of factors associated with HPIV positivity across all age groups are shown in Table 6. Participants who resided in Irbid or Zarqa had a lower likelihood of testing positive for HPIV than those in the capital (Amman). In addition, those living in the northern region had a lower likelihood of testing positive compared with the middle region, while those in the south had a higher likelihood. Furthermore, age showed a significant correlation, i.e., an increase in age increased the odds of being HPIV-positive. Individuals who were 6 months old or younger had significantly lower odds of testing positive than those who were older.

### 3.3. Predictors of Complications

Further regression analysis was conducted for the predictors of the presence of at least one complication (Table 7). This includes the definition of one complication: mortality, bacterial coinfection, respiratory distress requiring oxygen (either invasive or non-invasive), pneumonia, cardiovascular complications including heart failure or bradycardia or other cardiovascular complications, and respiratory failure.

Patients showed a significantly greater likelihood of having complications when co-infected with RSV or influenza and significantly lower odds when positive for HPIV only or negative for HPIV (*p*-value < 0.001 for both). Furthermore, male gender, a longer hospital stay, having asthma and the presence of clinical findings such as respiratory acidosis were factors increasing the likelihood of having at least one complication. Age and attending kindergarten were factors that decreased the odds of having at least one complication.

### 3.4. Comparing HPIV Types

HPIV-3 was identified most frequently. When HPIV-3-positive patients were compared with those positive for other HPIV types combined (due to a small number of HPIV-1, HPIV-2, and HPIV-4 cases) in terms of the presence of complications, there were statistically significant differences between HPIV-3 and the other types combined. When comparing the presence of symptoms between HPIV-3 and the other types combined, we found that those positive for HPIV-3 had statistically higher rates of nasal congestion (62.25% vs. 18.18%, *p*-value < 0.001) and tachypnea (52.94% vs. 13.64%, *p*-value < 0.001).

## 4. Discussion

This study provides valuable insights into the burden of human parainfluenza virus (HPIV) in children under five years of age in Jordan and highlights the clinical and geographic variability of the disease. By examining the association between clinical symptoms, co-infections, and geographic factors, our findings contribute to a more nuanced understanding of HPIV in this population.

Our results show that HPIV is a significant contributor to respiratory illnesses in children under two years of age, with almost 23% of children testing positive. A recent systematic review [4] of more than 200 studies showed that approximately 13% of acute respiratory tract infections are due to HPIVs. Our study only included those who were hospitalized, whereas that review also included participants with milder diseases in the community, which may account for the difference seen. Other studies have shown that up to 40% of children hospitalized with lower respiratory tract infections test positive for HPIV during periods of high circulation [1]. Our positivity rate of 23% is much higher than the previous rate of 7% that was obtained for Jordan, based on an analysis of samples collected from one hospital in Jordan between March 2010 and March 2013 [6]. This change is consistent with recent studies highlighting the epidemiological changes and increase in HPIV positivity after the COVID-19 pandemic [10,11,12].

Human Parainfluenza 3 was the most common serotype identified in our study. HPIV-3 is known to cause severe LRTIs, such as bronchiolitis and pneumonia, particularly in younger children. Our data showed higher rates of HPIV-3 compared with the above study from Jordan; in ours, HPIV-3 contributed to 90.3% of the total, while in the previous study, the number was lower (58%). Furthermore, a recent study from China also revealed that HPIV-3 is the most prevalent serotype (59.55%) [13]. Similar findings were reported from Turkey [14].

Among infected children, those presenting with symptoms such as a sore throat, rhinorrhea, nasal congestion, poor feeding and tachypnea were compared with those who tested negative for HPIV. In those who only tested positive for HPIV (with no co-infections), a sore throat and poor feeding were more common than in those who were HPIV negative. These findings are consistent with previous studies demonstrating that HPIV can lead to a wide spectrum of clinical manifestations, from mild upper respiratory tract infections to severe lower respiratory tract infections requiring hospitalization [15,16]. Notably, our study identified that HPIV-positive participants who had specific factors such as male gender, asthma and the presence of investigations such as respiratory acidosis had an increased likelihood of having at least one complication, emphasizing the need for early recognition and management in these cases. Previous studies [1] have shown that factors such as malnutrition, overcrowding, vitamin A deficiency, and exposure to environmental smoke or toxins predispose children to HPIV infections. In addition, gender and ethnicity also appear to play a role, as HPIV-associated bronchiolitis reportedly occurs more often in non-white males, which our findings corroborate.

Co-infections were common, similar to other studies [8,9], and co-infections with RSV or influenza were associated with significantly worse outcomes. Children with RSV or influenza co-infections were more likely to require mechanical ventilation. This finding aligns with a systematic review that suggested that co-infections with HPIV and RSV are associated with a higher risk of intensive care unit admission in young children compared with just RSV infection [17]. The synergistic impact of HPIV and other respiratory viruses underscores the importance of accurate and timely diagnosis in managing co-infections to reduce the risk of complications.

Geographic differences in HPIV positivity rates and disease severity were evident across the regions studied. Children from Amman exhibited higher rates of HPIV positivity and more severe disease compared with those from either Irbid or Zarqa. These differences may be attributed to variations in environmental factors, such as the population density, air quality, and climate, which can influence viral transmission and immune responses [18]. Positivity rates were higher in the south of Jordan, which has a higher poverty rate than other regions in Jordan [19]. Additionally, socioeconomic disparities, including access to healthcare and vaccination coverage, may contribute to the observed geographic variability. Understanding these regional differences is essential for tailoring public health interventions and allocating healthcare resources effectively.

From a public health perspective, our findings underscore the need for the enhanced surveillance of HPIV and other respiratory viruses in children under five years old. The routine monitoring of regional and seasonal trends could inform vaccination strategies and public health campaigns aimed at reducing transmission. The development of effective vaccines against HPIV remains a priority, particularly given the increased risk of severe outcomes in co-infected children and those from vulnerable geographic regions.

Improved monitoring systems would allow for the timely identification of regional and seasonal trends, enabling healthcare systems to respond more effectively to outbreaks. These data could also guide public health campaigns that aim to reduce viral transmission, such as promoting hygiene measures, educating caregivers about early symptoms, and emphasizing the importance of seeking medical care for respiratory illnesses in young children. Additionally, such surveillance would provide a foundation for developing and implementing targeted vaccination strategies.

The development of effective vaccines against HPIV remains an urgent priority [20]. A vaccine targeting HPIV could significantly reduce the healthcare burden, particularly in regions with a high prevalence of disease. Vulnerable populations, including those in densely populated or socioeconomically disadvantaged areas, would especially benefit from vaccination programs. Beyond vaccine development, integrating these efforts with broader respiratory virus prevention strategies could reduce morbidity, mortality, and the overall healthcare impact of HPIV in young children.

## 5. Limitations

This study included a large sample of 1000 participants from representative sites in Jordan and presented data on HPIV with and without coinfections, along with data on the four serotypes. However, this study has several limitations; the main limitation is that it was conducted during the winter respiratory virus season and over a relatively short duration (four months) between November and March. Due to budget constraints, we could not recruit throughout the whole year. The seasonality of HPIV is different to RSV and influenza; thus, if we had recruited all year, we may have found differences in the HPIV epidemiology. Also, the data are based on inpatients, without including children from other settings such as emergency departments and general practice clinics. Therefore, the results are focused on severe cases requiring admission. Future studies should also assess the epidemiological and clinical burden of HPIV in these settings where children’s clinical presentations do not require hospital admission.

## 6. Conclusions

In conclusion, this study demonstrates that HPIV is a significant cause of respiratory illness in young children in Jordan, with the disease severity influenced by the presence of specific symptoms, co-infections, and geographic factors. Tailored public health interventions, the early recognition of severe symptoms, and comprehensive diagnostic strategies are essential for mitigating the impact of HPIV in this population. Further research is needed to explore the underlying mechanisms that drive geographic variability and to evaluate the long-term outcomes of children with severe HPIV infections.

## Figures and Tables

**Table 1:** Presence of symptoms by HPIV positivity among children below 5 years in Jordan (15 November 2022–14 April 2023).

	HPIV Results (*n* = 1000)		
Symptoms	HPIV Negative (*n* = 774)	HPIV Positive (Total) (*n* = 226)	
Count (Column%)	Count N (%)	Count N (%)	*p*-Value *
Fever	765 (98.84%)	223 (98.67%)	0.841
Cough	727 (93.93%)	211 (93.36%)	0.757
Sore throat	147 (18.99%)	57 (25.22%)	0.041
Rhinorrhea	392 (50.65%)	145 (64.16%)	<0.001
Nasal congestion	323 (41.73%)	131 (57.96%)	<0.001
Poor Feeding	361 (46.64%)	132 (58.41%)	0.002
Hypoxia/Cyanosis	218 (28.17%)	62 (27.43%)	0.829
Breathlessness	320 (41.34%)	108 (47.79%)	0.085
Respiratory crackles	437 (56.46%)	129 (57.08%)	0.869
Apnea > 10 s	7 (0.90%)	3 (1.33%)	0.574
Wheezing	365 (47.16%)	116 (51.33%)	0.270
Low activity level	414 (53.49%)	123 (54.42%)	0.804
Tachypnea	241 (31.14%)	101 (44.69%)	<0.001
Post Tussive Vomiting	258 (33.33%)	83 (36.73%)	0.344

* Chi-square test, statistically significant at *p* < 0.05. *p*-value is the result of testing the difference between patients negative and positive for HPIV (total).

**Table 2:** The presence of symptoms by HPIV positivity and the presence of coinfection with RSV and/or influenza among children below 5 years in Jordan (15 November 2022–14 April 2023).

	HPIV Results (*n* = 1000)				
Symptoms	HPIV Negative (*n* = 774)	HPIV Positive Only (*n* = 126)	HPIV Positive with Coinfection ** (*n* = 100)		
Count (Column%)	Count N (%)	Count N (%)	Count N (%)	*p*-Value *	*p*-Value ^
Fever	765 (98.84%)	124 (98.41%)	99 (99.00%)	0.904	0.702
Cough	727 (93.93%)	114 (90.48%)	97 (97.00%)	0.124	0.050
Sore throat	147 (18.99%)	38 (30.16%)	19 (19.00%)	0.015	0.055
Rhinorrhea	392 (50.65%)	78 (61.90%)	67 (67.00%)	0.001	0.428
Nasal congestion	323 (41.73%)	73 (57.94%)	58 (58.00%)	<0.001	0.992
Poor Feeding	361 (46.64%)	76 (60.32%)	56 (56.00%)	0.006	0.513
Hypoxia/Cyanosis	218 (28.17%)	33 (26.19%)	29 (29.00%)	0.876	0.638
Breathlessness	320 (41.34%)	54 (42.86%)	54 (54.00%)	0.055	0.096
Respiratory crackles	437 (56.46%)	71 (56.35%)	58 (58.00%)	0.956	0.803
Apnea > 10 s	7 (0.90%)	1 (0.79%)	2 (2.00%)	0.567	0.431
Wheezing	365 (47.16%)	63(50.00%)	53 (53.00%)	0.492	0.654
Low activity level	414 (53.49%)	75 (59.52%)	48 (48.00%)	0.219	0.084
Tachypnea	241 (31.14%)	56 (44.44%)	45 (45.00%)	0.001	0.933
Post Tussive Vomiting	258 (33.33%)	46 (36.51%)	37 (37.00%)	0.637	0.939

* Chi-square test, statistically significant at *p* < 0.05. *p*-value is the result of testing the differences between patients who are HPIV negative, HPIV positive only and HPIV positive with coinfection. ^ Chi-square test, statistically significant at *p* < 0.05. *p*-value is the result of testing the differences between patients who are HPIV positive only and HPIV positive with coinfection, without including HPIV-negative cases in the analysis. ** coinfection refers to RSV or being positive for influenza and HPIV.

**Table 3:** Duration of symptoms by HPIV positivity among children below 5 years in Jordan (15 November 2022–14 April 2023).

	HPIV Result (*n* = 1000)				
Symptoms	Negative		Positive		*p*-Value *
	Mean	Standard Deviation	Mean	Standard Deviation	
Fever (days)	4.017	3.687	3.765	3.888	0.370
Cough (days)	5.403	5.110	5.628	7.071	0.596
Sore throat (days)	0.925	2.810	1.186	2.840	0.221
Rhinorrhea (days)	2.886	4.202	3.633	4.845	0.024
Nasal congestion (days)	2.253	3.975	3.606	6.004	<0.001
Poor Feeding (days)	1.680	2.751	2.044	2.742	0.081
Hypoxia/Cyanosis (days)	1.014	2.727	1.058	4.490	0.858
Breathlessness (days)	1.618	2.783	1.712	2.760	0.652
Respiratory crackles (days)	2.429	3.432	2.111	2.747	0.201
Apnea > 10 s (days)	0.253	1.721	0.119	0.659	0.253
Wheezing (days)	1.594	2.638	1.867	2.810	0.178
Low activity level (days)	2.005	2.948	1.748	2.169	0.223
Tachypnea (days)	1.037	2.094	1.469	2.361	0.008
Post Tussive Vomiting (days)	1.059	2.093	1.026	1.916	0.832

* *t*-test statistically significant at *p* < 0.05.

**Table 4:** Descriptive statistics for signs and clinical findings by HPIV positivity among children below 5 years in Jordan (15 November 2022–14 April 2023).

		HPIV (*n* = 1000)		
		HPIV Negative (*n* = 774)	HPIV Positive (Total) (*n* = 226)	
Clinical Finding		Count N (%)	Count N (%)	*p*-Value *
Chest X-ray infiltrate		496 (64.08%)	151 (66.81%)	0.751
White blood cell count (×10^9^/L)	<4.0	10 (1.29%)	5 (2.21%)	0.465
	>10.0	489 (63.18%)	139 (61.50%)	
	4 to 10	275 (35.53%)	82 (36.28%)	
Other Clinical Manifestations:				
• Apnea > 10 s		7 (0.90%)	3 (1.33%)	0.574
• Dehydration		195 (25.19%)	38 (16.81%)	0.009
• Hypoxia (SpO_2_ < 92%)		185 (23.90%)	49 (21.68)	0.488
• Subcostal/intercostal retractions		331 (42.76%)	100 (44.25%)	0.692
• Wheezes as a sign		424 (54.78%)	142 (62.83%)	0.032
• Tachypnea as a sign		384 (49.61%)	130 (57.52%)	0.036
• Cyanosis as a sign		62 (8.01%)	16 (7.08%)	0.646
• Acute respiratory distress		212 (27.39%)	70 (30.97%)	0.292
• Required ICU admission		72 (9.30%)	24 (10.62%)	0.554
• Overall oxygen needs Non-invasive ventilation		42 (5.43%)	17 (7.52%)	0.239
• Overall oxygen needs invasive ventilation		6 (0.78%)	3 (1.33%)	0.439

* Chi-square test, statistically significant at *p* < 0.05. *p*-value is the result of testing the difference between patients who are negative and positive for HPIV (total).

**Table 5:** Descriptive statistics for signs and clinical findings by HPIV positivity and the presence of coinfection with RSV and/or influenza among children below 5 years in Jordan (15 November 2022–14 April 2023).

		HPIV (*n* = 1000)				
		HPIV Negative (*n* = 774)	HPIV Positive Only (*n* = 126)	HPIV Positive with Coinfection ** (*n* = 100)		
Clinical Finding		Count N (%)	Count N (%)	Count N (%)	*p*-Value *	*p*-Value ^
Chest X ray infiltrate		496 (64.08%)	84 (66.67%)	67(67.00%)	0.751	0.958
White blood cell count (×10^9^/L)	<4.0	10 (1.29%)	3 (2.38%)	2 (2.00%)	0.338	0.281
	>10.0	489 (63.18%)	83 (65.87%)	56 (56.00%)		
	4 to 10	275 (35.53%)	40 (31.75%)	42 (42.00%)		
Other Clinical Manifestations:						
Apnea > 10 s		7 (0.90%)	1 (0.79%)	2 (2.00%)	0.567	0.431
Dehydration		195 (25.19%)	18 (14.29%)	20 (20.00%)	0.019	0.254
Hypoxia (SpO_2_ < 92%)		185 (23.90%)	22 (17.46%)	27 (27.00%)	0.191	0.084
Subcostal/intercostal retractions		331 (42.76%)	54(42.8%)	46(46.00%)	0.826	0.637
Wheezes as a sign		424 (54.78%)	76 (60.32%)	66 (66.00%)	0.069	0.380
Tachypnea as a sign		384 (49.61%)	72 (57.14%)	58 (58.00%)	0.111	0.897
Cyanosis as a sign		62 (8.01%)	6 (4.76%)	10 (10.00%)	0.311	0.127
Acute respiratory distress		212 (27.39%)	41 (32.54%)	29 (29.00%)	0.483	0.568
Required ICU admission		72 (9.30%)	13 (10.32%)	11 (11.00%)	0.827	0.869
Overall oxygen needs Non-invasive ventilation		42 (5.43%)	10 (7.94%)	7 (7.00%)	0.479	0.791
Overall oxygen needs invasive ventilation		6 (0.78%)	0 (0.00%)	3 (3.00%)	0.045	0.050

* Chi-square test, statistically significant at *p* < 0.05. *p*-value is the result of testing the differences between patients who are HPIV negative, HPIV positive only and HPIV positive with coinfection. ^ Chi-square test, statistically significant at *p* < 0.05. *p*-value is the result of testing the differences between patients who are HPIV positive only and HPIV positive with coinfection, without including HPIV-negative cases in the analysis. ** coinfection refers to RSV or being positive for influenza and HPIV.

**Table 6:** Binary logistic regression analysis of factors associated with an HPIV-positive result across all age groups among children below 5 years in Jordan (15 November 2022–14 April 2023).

	Coefficient	*p*-Value	OR *	CI 95%
City				
Irbid	−0.633	0.005	0.53	(0.34–0.82)
Zarqa	−0.464	0.033	0.63	(0.41–0.96)
Karak	0.159	0.426	1.17	(0.79–1.73)
Amman	reference			
Age (months)	0.02	<0.001	1.02	(1.013–1.029)
Age ≤ 6 months				
YES	−0.461	0.005	0.631	(0.458–0.868)
NO	Reference			
Parents COVID Vaccine uptake				
YES	−0.497	0.046	0.608	(0.373–0.991)
NO	reference			
Father COVID Vaccine Uptake				
YES	−0.57	0.024	0.565	(0.34–0.93)
NO	Reference			
Breastfeed				
Exclusive	0.393	0.033	1.483	(1.033–2.128)
Mixed	0.143	0.458	1.154	(0.790–1.685)
NO	Reference			
Wheezing clinical symptom present				
YES	0.38	0.012	1.46	(1.09–1.96)
NO	reference			
Tachypnea clinical symptom present				
YES	0.581	<0.001	1.78	(1.32–2.41)
NO	reference			
Nasal congestion clinical symptom present				
YES	0.655	<0.001	1.92	(1.426–2.599)
NO	Reference			
Nasal congestion duration	0.057	<0.001	1.06	(1.026–1.092)
Rhinorrhea symptom				
YES		<0.001	1.74	(1.284–2.369)
NO	reference			
Rhinorrhea days	0.556	0.025	1.04	(1.004–1.069)
Poor feeding clinical symptom				
YES	0.474	0.002	1.61	(1.19–2.17)
NO	reference			
Dehydration				
YES	−0.51	0.009	0.60	(0.409–0.881)
NO	reference			
Sore throat				
YES	0.363	0.042	1.44	(1.014–2.041)
NO	reference			
Surfactant given at birth				
YES	0.0461	0.015	1.59	(1.092–2.304)
NO	reference			

* Unadjusted odds ratio.

**Table 7:** Binary logistic regression analysis of the factors associated with the presence of complications across all age groups Among children below 5 years in Jordan (15 November 2022–14 April 2023).

	Coefficient	OR	*p*-Value	CI 95%
Infection				
Parainfluenza without coinfection	−4.068	0.017	<0.001	(0.004–0.073)
Parainfluenza with coinfection **	0.954	2.597	<0.001	(1.555–4.337)
Parainfluenza Negative	Reference *			
Age (months)	−0.064	0.938	<0.001	(0.926–0.951)
Gender (Male)	0.395	1.485	0.018	(1.069–2.062)
Total length of hospital stay	0.072	1.075	0.017	(1.013–1.14)
Asthma	1.100	3.004	0.006	(1.365–6.608)
Presence of respiratory acidosis	0.793	2.21	0.017	(1.153–4.235)
Patient attending kindergarten	−0.985	0.373	0.004	(0.192–0.728)
Constant	−1.672	0.188	<0.001	(0.123–0.287)

* The reference category in all binary variables was assigned to “No” as a reference group. ** coinfection refers to being positive for RSV or influenza.

## Data Availability

The original contributions presented in the study are included in the article/Supplementary Materials; further inquiries can be directed to the corresponding author.

## Supplementary Materials

The following supporting information can be downloaded at: https://www.mdpi.com/article/10.3390/v17020170/s1. Table S1: HPIV positivity by month of Admission Among Children Below 5 years in Jordan (15 November 2022–14 April 2023); Table S2: Investigating Demographic Factors and Medical Histories Associated with HPIV results Among Children Below 5 years in Jordan (15 November 2022–14 April 2023).

## References

[1] Branche A.R., Falsey A.R. (2016). Parainfluenza Virus Infection. Semin. Respir. Crit. Care Med..

[2] Li Y., Reeves R.M., Wang X., Bassat Q., Brooks W.A., Cohen C., Moore D.P., Nunes M., Rath B., Campbell H. (2019). Global Patterns in Monthly Activity of Influenza Virus, Respiratory Syncytial Virus, Parainfluenza Virus, and Metapneumovirus: A Systematic Analysis. Lancet Glob. Health.

[3] Henrickson K.J. (2003). Parainfluenza Viruses. Clin. Microbiol. Rev..

[4] Wang X., Li Y., Deloria-Knoll M., Madhi S.A., Cohen C., Arguelles V.L., Basnet S., Bassat Q., Brooks W.A., Echavarria M. (2021). Global Burden of Acute Lower Respiratory Infection Associated with Human Parainfluenza Virus in Children Younger than 5 Years for 2018: A Systematic Review and Meta-Analysis. Lancet Glob. Health.

[5] Wang C., Liu J., Mi Y., Chen J., Bi J., Chen Y. (2021). Clinical Features and Epidemiological Analysis of Respiratory Human Adenovirus Infection in Hospitalized Children: A Cross-Sectional Study in Zhejiang. Virol. J..

[6] Howard L.M., Rankin D.A., Spieker A.J., Gu W., Haddadin Z., Probst V., Rahman H., McHenry R., Pulido C.G., Williams J.V. (2021). Clinical Features of Parainfluenza Infections among Young Children Hospitalized for Acute Respiratory Illness in Amman, Jordan. BMC Infect. Dis..

[7] Abu-Helalah M., Al-Shatnawi S.F., Abu Lubad M., Al-Zayadneh E., Jdaitawi H., Harahsheh M., AL-Iede M., Nafi O., Yousef R., Almaaitah I. (2024). The Epidemiology, Clinical, and Economic Burdens of Respiratory Syncytial Virus Infections Amongst Hospitalized Children Under 5 Years of Age in Jordan: A National Multi-Center Cross-Sectional Study. Viruses.

[8] Gao Y., Ma Y., Feng D., Zhang F., Wang B., Liu X., Zhu B., Xie H., Zhao L., Long X. (2024). Epidemiological Characteristics of Human Parainfluenza Viruses Infections—China, 2019-2023. China CDC Wkly..

[9] Rafeek R., Divarathna M., Morel A., Noordeen F. (2021). Epidemiological and Clinical Characteristics of Children with Human Parainfluenza Virus Associated Acute Respiratory Infection in a General Hospital in Sri Lanka. J. Clin. Virol. Plus.

[10] Li L., Jia R., Zhang Y., Sun H., Ma J. (2023). Changes of Parainfluenza Virus Infection in Children before and after the COVID-19 Pandemic in Henan, China. J. Infect..

[11] Xu X., Zhang Y., Xu L., Jiang W., Hao C. (2024). Analysis of Respiratory Pathogen Detection in Hospitalized Children with Acute Respiratory Tract Infections after Ending the Zero COVID Policy. Sci. Rep..

[12] Cohen S., Dabaja-Younis H., Etshtein L., Gnatt I., Szwarcwort-Cohen M., Hadash A., Kassis I., Halberthal M., Shachor-Meyouhas Y. (2024). Burden of Viral Respiratory Infections in the Pediatric Intensive Care Unit: Age, Virus Distribution, and the Impact of the COVID-19 Pandemic. Eur. J. Pediatr..

[13] Xiao M., Banu A., Zeng X., Shi S., Peng R., Chen S., Ge N., Tang C., Huang Y., Wang G. (2024). Epidemiology of Human Parainfluenza Virus Infections among Pediatric Patients in Hainan Island, China, 2021–2023. Pathogens.

[14] Bicer S., Giray T., Çöl D., Erdağ G.Ç., Vitrinel A., Gürol Y., Çelik G., Kaspar C., Küçük Ö. (2013). Virological and Clinical Characterizations of Respiratory Infections in Hospitalized Children. Ital. J. Pediatr..

[15] Hall C.B. (2001). Respiratory Syncytial Virus and Parainfluenza Virus. N. Engl. J. Med..

[16] Liu W.-K., Liu Q., Chen D.-H., Liang H.-X., Chen X.-K., Huang W.-B., Qin S., Yang Z.-F., Zhou R. (2013). Epidemiology and Clinical Presentation of the Four Human Parainfluenza Virus Types. BMC Infect. Dis..

[17] Li Y., Pillai P., Miyake F., Nair H. (2020). The Role of Viral Co-Infections in the Severity of Acute Respiratory Infections among Children Infected with Respiratory Syncytial Virus (RSV): A Systematic Review and Meta-Analysis. J. Glob. Health.

[18] Xu B., Wang J., Li Z., Xu C., Liao Y., Hu M., Yang J., Lai S., Wang L., Yang W. (2021). Seasonal Association between Viral Causes of Hospitalised Acute Lower Respiratory Infections and Meteorological Factors in China: A Retrospective Study. Lancet Planet. Health.

[19] Alalaya M.M., Alsoboa S.S., Khattab S.A.A. (2015). Poverty Effects of House Holds in the Southern Region of Jordan. Eur. J. Bus. Manag..

[20] Afroz S., Saul S., Dai J., Surman S., Liu X., Park H.-S., Le Nouën C., Lingemann M., Dahal B., Coleman J.R. (2024). Human Parainfluenza Virus 3 Vaccine Candidates Attenuated by Codon-Pair Deoptimization Are Immunogenic and Protective in Hamsters. Proc. Natl. Acad. Sci. USA.

