# Supplementary material for: The Epidemiology and Clinical Burdens of Human Parainfluenza Virus Infections Amongst Hospitalized Children Under 5 Years of Age in Jordan: A National Multi-Center Cross-Sectional Study
Source: Viruses. 2025 Jan 25;17(2):170. doi: 10.3390/v17020170 (PMC11861330; doi:10.3390/v17020170)
Supplement: Supplementary file 1 [file viruses-17-00170-s001.zip › viruses-3451170-supplementary.pdf]

Supplementary Table S1. HPIV positivity by month of Admission Among Children Below 5 years in Jordan (15 November 2022 - 14 April 2023).

			HPIV		Total	P-value*
			NO	YES		
**Date month**	Nov-2022	Count	307	13	320	<0.001
		% within Month	95.94%	4.06%	100%	
	Dec-2022	Count	312	144	456	
		% within Month	68.42%	31.58%	100%	
	Jan-2023	Count	94	54	148	
		% within Month	63.51%	36.49%	100%	
	Feb-2023	Count	31	9	40	
		% within Month	77.5%	22.5%	100%	
	Mar-2023	Count	30	6	36	
		% within Month	83.33%	16.67%	100%	

***Chi-square, statistically significant at P < 0.05.**

Supplementary Table s2: Investigating Demographic Factors and Medical Histories Associated with HPIV results Among Children Below 5 years in Jordan (15 November 2022 - 14 April 2023)..

Characteristic	HPIV Result				P-value*
	Negative (N=774)		Positive (N=226)		
	Count	Row (N %)	Count	Row (N %)	
**Age in months, mean (SE)**	15.77 (0.58)		21.65 (1.15)		**<0.001**
**Age in months, median, [Q1-Q3]**	8.26 (2.83- 26.3)		18.8 (4.56- 34.56)		
**Age ≤ 6 months**					
**NO**	464	74.48%	159	25.52%	**0.005**
**YES**	310	82.23%	67	17.77%	
**Gender, n (%)**					
**Female patients**	324	78.26%	90	21.74%	0.584
**Male patients**	450	76.79%	136	23.21%	
**Region, n (%)**					
· **Middle**	388	77.60%	112	22.40%	**<0.001**
· **North**	210	84.00%	40	16.00%	
· **South**	176	70.40%	74	29.60%	
**City, n (%)**					
· **Amman**	184	73.60%	66	26.40%	**<0.001**
· **Zarqa**	204	81.60%	46	18.40%	
· **Irbid**	210	84.00%	40	16.00%	

· **Karak**		176	70.40%	74	29.60%	
**Living area, n (%)**						
· **Rural**		251	76.76%	76	23.24%	0.735
· **Urban**		523	77.71%	150	22.29%	
**Admission through, n (%)**						
· **ER referral**		95	83.33%	19	16.67%	0.206
· **ER without referral**		599	77.09%	178	22.91%	
· **GP**		5	83.33%	1	16.67%	
· **Outpatient clinic**		70	71.43%	28	28.57%	
· **Pediatrician**		5	100.00%	0	0.00%	
**Preterm or Full term**	Full term	650	77.11%	193	22.89%	0.606
	preterm	124	78.98%	33	21.02%	
**WGA (Weeks for Gestational Age)**	<29	1	50.00%	1	50.00%	0.158
	29-30	4	100.00%	0	0.00%	
	31-32	9	56.25%	7	43.75%	
	33-34	19	76.00%	6	24.00%	
	35-36	91	82.73%	19	17.27%	
	>=37	650	77.11%	193	22.89%	
**Delivery**	Normal VD,	414	77.38%	121	22.62%	0.989
	Caesarean	360	77.42%	105	22.58%	
**Meconium-stained liquor**	NO	758	77.19%	224	22.81%	0.24
	YES	16	88.89%	2	11.11%	
**NICU**	NO	585	77.28%	172	22.72%	0.871
	YES	189	77.78%	54	22.22%	
**NICU ventilation**	NO	674	78.10%	189	21.90%	0.184
	YES	100	72.99%	37	27.01%	
**Surfactant Given**	NO	659	78.83%	177	21.17%	**0.015**
	YES	115	70.12%	49	29.88%	
**Breastfed**	NO	269	80.54%	65	19.46%	0.09
	Exclusive	254	73.62%	91	26.38%	
	Mixed	251	78.19%	70	21.81%	
**Mother smoking during pregnancy**	NO	735	77.21%	217	22.79%	0.513
	YES	39	81.25%	9	18.75%	
**Patient on special milk/diet**	NO	745	77.12%	221	22.88%	0.263
	YES	29	85.29%	5	14.71%	
**Overcrowding**	NO	646	77.18%	191	22.82%	0.142
	YES	128	78.53%	35	21.47%	
**Parents smoking cigarettes or Shisha**	NO	215	77.62%	62	22.38%	0.919
	YES	559	77.32%	164	22.68%	
	NO	597	77.33%	175	22.67%	0.924

**Smoking inside home by parents or other households**	YES	177	77.63%	51	22.37%	
**Patient regular medications**	NO	632	77.74%	181	22.26%	0.595
	YES	142	75.94%	45	24.06%	
**Highest completed education of mother**	Diploma school	72	80.90%	17	19.10%	0.686
	Primary school	85	77.98%	24	22.02%	
	Secondary school	384	78.21%	107	21.79%	
	University BSc	214	75.35%	70	24.65%	
	University Postgraduate	19	70.37%	8	29.63%	
**Highest completed education of Father**	Diploma school	65	81.25%	15	18.75%	0.256
	Primary school	107	78.68%	29	21.32%	
	Secondary school	388	78.38%	107	21.62%	
	University BSc	179	75.85%	57	24.15%	
	University Postgraduate	35	66.04%	18	33.96%	
**Chronic conditions:**						
**Asthma**	NO	735	77.53%	213	22.47%	0.67
	YES	39	75.00%	13	25.00%	
**Bronchopulmonary dysplasia**	NO	773	77.38%	226	22.62%	0.589
	YES	1	100.00%	0	0.00%	
**Congenital heart disease**	No	744	77.66%	214	22.34%	0.344
	YES	30	71.43%	12	28.57%	
**Neuromuscular disease**	NO	764	77.48%	222	22.52%	0.591
	YES	10	71.43%	4	28.57%	
**Other comorbidities****	NO	692	77.93%	196	22.07%	0.261
	YES	82	73.21%	30	26.79%	
**Cystic fibrosis**	NO	773	77.38%	226	22.62%	0.589
	YES	1	100.00%	0	0.00%	
**Other congenital disease*****	NO	766	77.53%	222	22.47%	0.371
	YES	8	66.67%	4	33.33%	
**Eczema (Atopy)**	NO	759	77.69%	218	22.31%	
	YES	15	65.22%	8	34.78%	

**Patient Chronic Conditions**	NO	617	78.90%	165	21.10%	**0.032**
	YES	157	72.02%	61	27.98%	
**Patient attending kindergarten or day care**	NO	700	77.01%	209	22.99%	0.349
	YES	74	81.32%	17	18.68%	
**Parent with history of atopic eczema**	NO	718	78.13%	201	21.87%	0.064
	YES	56	69.14%	25	30.86%	
**Siblings attending kindergarten or daycare**	NO	619	77.09%	184	22.91%	0.631
	YES	155	78.68%	42	21.32%	
**Parent with history of asthma**	NO	715	78.06%	201	21.94%	0.101
	YES	59	70.24%	25	29.76%	
**Siblings with history of asthma**	NO	712	77.56%	206	22.44%	0.686
	YES	62	75.61%	20	24.39%	
**Siblings <5 years living in the same household**	NO	307	75.25%	101	24.75%	0.176
	YES	467	78.89%	125	21.11%	

*Chi-square test, statistically significant at P < 0.05. for Age t-test was used.
** Other comorbidities: including congenital heart disease, Ventricular Septal Defect (VSD), Antenatally Diagnosed Congenital Pulmonary Airway Malformation (CPAM) Type 1 with extra lobar sequestration, and Complete Atrioventricular (AV) Canal, along with Atrial Septal Defect (ASD) and VSD leading to heart failure. Additionally, Down syndrome, Global Developmental Delay (GDD), and epilepsy. Other conditions include Reactive Airway Disease (RAD), Retinopathy of Prematurity (ROP), renal anomalies, seizures and Glucose-6-Phosphate Dehydrogenase Deficiency (G6PD).
***In terms of other congenital diseases, there is a chest deformity, Gastroesophageal Reflux Disease (GERD), Pierre Robin Syndrome, horseshoe kidney, laryngomalacia, single kidney, single lung, hydrocephalus, and Vesicoureteral Reflux (VUR).
